# High Anti-Reflection Large-Scale Cup-Shaped Nano-Pillar Arrays via Thin Film Anodic Aluminum Oxide Replication

**DOI:** 10.3390/nano12111875

**Published:** 2022-05-30

**Authors:** Tangyou Sun, Furong Shui, Xiancui Yang, Zhiping Zhou, Rongqiao Wan, Yun Liu, Cheng Qian, Zhimou Xu, Haiou Li, Wenjing Guo

**Affiliations:** 1 Guangxi Key Laboratory of Precision Navigation Technology and Application, Guilin University of Electronic Technology, Guilin 541004, China; suntangyou@guet.edu.cn (T.S.); sfr1277353567@163.com (F.S.); yangxiancui2022@163.com (X.Y.); 18870283690@163.com (Y.L.); lihaiou@guet.edu.cn (H.L.); wenjing@guet.edu.cn (W.G.); 2 State Key Laboratory of Advanced Optical Communication Systems and Networks, School of Electronics Engineering and Computer Science, Peking University, Beijing 100091, China; zjzhou@pku.edu.cn; 3 PerkinElmer Management (Shanghai) Co., Ltd., Shanghai 201203, China; cheng.qian@perkinelmer.com; 4 School of Optical and Electronic Information, Huazhong University of Science and Technology, Wuhan 430074, China

**Keywords:** anodic aluminum oxide, light trapping, anti-reflection, thin film, solar cells

## Abstract

Surface anti-reflection (AR) with nanometer-scaled texture has shown excellent light trapping performance involving optical devices. In this work, we developed a simple and lithography-free structure replication process to obtain large scale surface cup-shaped nano-pillar (CSNP) arrays for the first time. A method of depositing was used for pattern transfer based on PMMA pre-coated through-hole anodic aluminum oxide (AAO) thin film (~500 nm), and eventually, the uniformity of the transferred nanostructures was guaranteed. From the spectrum (250 nm~2000 nm) dependent measurements, the CSNP nanostructured Si showed excellent AR performance when compared with that of the single-polished Si. Moreover, the CSNP was found to be polarization insensitive and less dependent on incidence angles (≤80°) over the whole spectrum. To further prove the excellent antireflective properties of the CSNP structure, thin film solar cell models were built and studied. The maximum value of J_ph_ for CSNP solar cells shows obvious improvement comparing with that of the cylinder, cone and parabola structured ones. Specifically, in comparison with the optimized Si_3_N_4_ thin film solar cell, an increment of 54.64% has been achieved for the CSNP thin film solar cell.

## 1. Introduction

At present, photoelectric devices can be seen everywhere, and have become an indispensable part of our everyday lives [1,2]. However, there are some limits that prevent the crucial properties of photoelectric devices from reaching the best effectiveness [3,4,5,6]. One of the most prominent problems is that photoelectric devices cannot make full use of sunlight because of the wavelength-dependent surface reflections, which greatly block the development of corresponding applications [7,8,9,10]. Therefore, the nanostructure-based anti-reflection (AR) technique emerged and has been playing an essential role ever since [11,12]. To date, although various nanostructured optoelectronic components and devices like solar cells [13,14,15,16], light-emitting diodes [17,18,19,20], and photodetectors [21,22] have been studied, the requirement of large area fabrication with nanometer-scale feature dimension FD (FD < *λ*/n, where *λ* is the light wavelength and n is the refractive index of the material under consideration) presents a bottle neck in its study and corresponding applications [17,23,24,25].

Inspired by the anodic aluminum oxide (AAO) pattern fabrication process [26], self-assembled nanostructure replication method has been proven to be an effective nanoscale solution for lithography-free, low cost, large area, and high throughput [24,27,28,29]. The excellent properties of self-assembled nanostructures can be obtained by large area adjustment of the geometric appearance; for example, the pore size, hole spacing, etc., can be continuously adjusted from tens of nanometers to hundreds of nanometers [24,30,31,32]. In addition, the equipment is simple, the process is mature and stable, and there is no upper limit of area [32,33,34,35]. These advantages make self-assembled pattern transfer a highly competitive method for large-area fabrication of nanostructures [24,33,34,35,36]. Zhiyong Fan et al. proposed a hole-shaped AAO template to replicate the nano-cone pillar-shaped PI substrate and then directly fabricated a thin film of a-Si:H solar cells on it. Compared with the devices fabricated on commercial flat PI substrates, the PEC of the nano-cone devices outperform by 48% [37]. Zhiqiang Yu et al. successfully prepared a 2-inch nano/micro hybrid AAO template through a “grain size” dependent Si-based ultra-thin Al anodic oxidation scheme. The transmittance of the replicated nano/micro hybrid PDMS was 42% higher than that of the nano-structured one; moreover, the AR enhancement of the nano/micro structured LED can reach as high as three folds or more [24]. Zhimou Xu et al. have successfully substantiated a simple and convenient technique to fabricate large-scale nanoarrays on GaN substrate by using non-through-hole AAO membrane (>1 μm) via the two-step ICP etching. The patterned GaN wafer shows 3.4-fold increase in PL intensity compared to the un-patterned one [38]. However, there are some shortcomings of existing methods: (1) the transfer from nanostructures to substrate cannot succeed by the polymer filling method, because of the uneven surface of micrometer-scale AAO [23,24,25]; (2) nanopattern transfer via AAO film thicker than 1 μm will lead to serious loss of feature size, inducing from the non-vertical side-wall of the AAO nano-holes [38,39]. During pattern transfer processes, especially when feature dimension goes down to dozens of nanometers, a cover mask should be as thin as possible for the purpose of accuracy pattern transfer (to avoid the loss of feature size), which however conflicts with the fragile nature of AAO (Al_2_O_3_). In this paper, we used an AAO thin film (~500 nm) depositing method to obtain a large-scale and uniform surface cup-shaped nano-pillar (CSNP) structure. The formation mechanism of the CSNP was discussed and the AR performance of the CSNP was studied based on incidence angles, wavelengths and polarizations. With light as the main medium of solar cells, the light reflection loss at the interface between high and low refractive index is inevitable, which is one of the main reasons for the low efficiency of solar cells at present. Nanostructured solar cells with advantages of high local electric field [8,40], wide operating wavelength [24,41], and omnidirectionality [24,41] have been expected as an ideal solution in recent years. Therefore, a thin film solar cell model was built to further study the light-trapping performance of the CSNP. The absorption of the CSNP-structured solar cell was greatly enhanced in the whole wavelength of 0.3–1.1 μm and showed even higher performance than the regular nanostructure (like cylinder, cone, and parabola) [27] solar cells. These results suggest that the CSNP could serve as an efficient AR structure and can be used to the application of high-performance optical components and devices.

## 2. Experimental

### 2.1. Pattern Replication via AAO/PMMA Thin Film-Based Depositing Method

**Figure 1:**
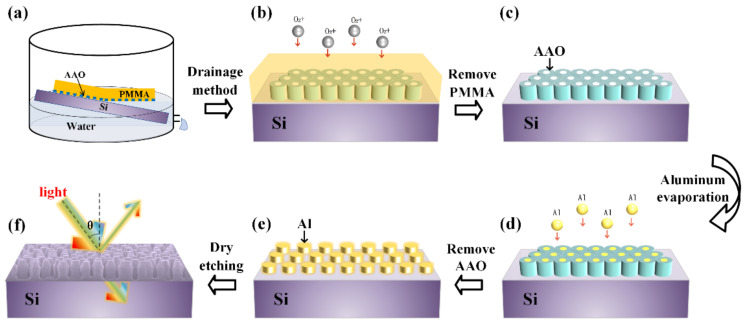
The schematic diagram of the AAO thin film-based pattern replication flow: (**a**,**b**), depositing method. (**b**,**c**), PMMA removal by oxygen dry etching. (**c**,**d**), aluminum evaporation. (**d**,**e**), remove AAO. (**e**,**f**), dry etching by using the aluminum etching mask.

Figure 1 is the fabrication flow of AAO thin film-based nanostructure pattern replication process. A commercially available through-hole AAO/PMMA thin film was used in the depositing method in Figure 1a. The free-standing through-hole AAO is too fragile to be handled especially when film thickness goes down to a few hundred nanometers, thus a hybrid AAO/PMMA structure was chosen for the purpose of large area applications. Before depositing, the AAO/PMMA thin film on substrate was carefully dipped into the DI water, leaving a free standing AAO/PMMA thin film floating on the surface. After that, an ultrasonic process was carried out for 30 s to level the thin film, and the bubbles underneath were able to be removed. A benefit of the soft and controllable depositing method, uniform AAO/PMMA thin film on silicon wafer can be obtained over a large area by controlling the leaning angle of the silicon substrate and the flow rate of the discharge liquid. The silicon substrate was pre-cleaned by piranha solution for 15 min to form a hydrophilic surface before putting into the depositing system. After the depositing, the PMMA/AAO/Si sample was naturally dried in air and the oxygen plasma over etching process (Figure 1b) was carried out to totally remove the upper PMMA support layer, resulting in a uniform through-hole AAO thin film on the surface of silicon as shown in Figure 1c. Then, the through-hole AAO structure was used as a shadow mask for aluminum evaporation, which means the aluminum will fill the empty space of AAO structure, as shown in Figure 1d. The evaporation was carried out in a thermal evaporation system (TRUMP-ZZ400D, Suzhou Trump Vacuum Technology, Suzhou, China) with a power of 200 w. Next, the AAO thin film was removed by pressing high temperature adhesive tape or 3M470 electroplated tape on the AAO thin film and enabling the tape to make full contact with AAO thin film, then remove the tape softly so the wanted Al structure can be available on the Si substrate, as shown in Figure 1e. Finally, the reactive ion etching (RIE, STRIPER-100, Beijing Zhongke Tailong Electronic Technology, Beijing, China) with a mixed gas of 60 sccm CF_4_ and 20 sscm Ar, a power of 75 w, was carried out for silicon dry etching. The etching rates for Al and Si were about 26.7 nm/min and 2.7 nm/min, respectively. Figure 1f is the surface morphology of the Si nano-arrays after removal of the Al pillar by H_3_PO_4_ solution.

### 2.2. Characterizations

The surface morphology and cross-sectional images of silicon nanostructures were measured by scanning electron microscope (SEM, JSM-IT500HR, JEOL, Tokyo, Japan). The reflectance of silicon nanostructures was measured by ultraviolet-visible Spectrophotometers (UV-VIS, Lambda 1050, PerkinElmer, Waltham, MA, USA) which were equipped with integrating sphere and angle adjusting systems. Specifically, the integrating sphere was used for total and diffuse reflectance measurements and the angle adjusting system was used for mirror reflectance analysis.

## 3. Result and Discussion

### 3.1. Nanostructured Surfaces via AAO Thin Film Replication Process

**Figure 2:**
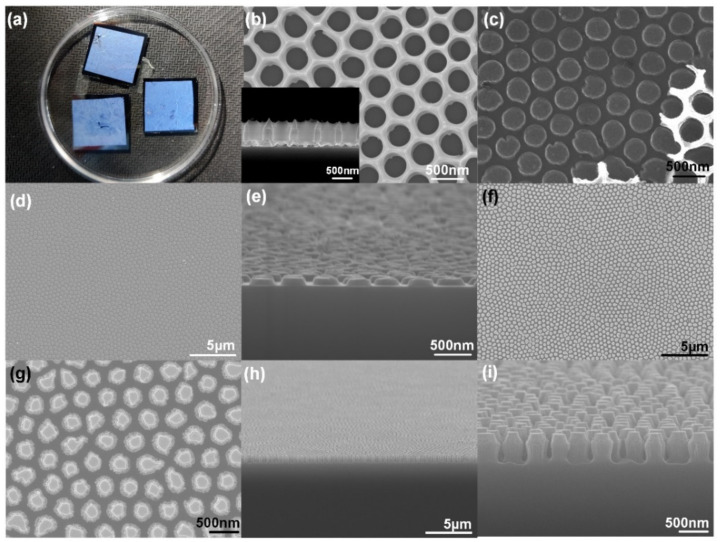
The main surface morphologies during the experiments: (**a**) Photography picture of the transferred through-hole AAO thin film on Si, the size of the silicon is 18 mm × 18 mm. SEM images for (**b**) top view of through-hole AAO thin film on Si (the picture of lower left corner is the side view of through-hole AAO thin film on Si), (**c**) after aluminum evaporation, (**d**) aluminum nano-arrays after the removal of AAO, (**e**) oblique view of the aluminum nano-arrays, (**f**,**g**) top view of the final Si nanostructure after the removal of aluminum, (**h**,**i**) oblique view of the nano-arrays of the final Si nanostructure.

Figure 2 shows the main surface morphologies during the experiments. Several benefits of the support effect of the PMMA layer, the soft depositing and oxygen plasma dry etching processes, and the free standing through-hole AAO thin film can be obtained for a large area as shown in Figure 2a. The effective area of transferred AAO thin film is around 15 mm × 15 mm in Figure 2a. Figure 2b is the SEM image of the transferred through-hole AAO thin film on Si, and the picture in the lower left corner is a sectional view of the AAO thin film on Si, the period of the through-hole AAO is around 479 nm, the aperture is around 363 nm, the thickness of the through-hole AAO is around 540 nm, and the thickness of the through-hole AAO was chosen under the following considerations: (1) the hole inside the AAO is not absolutely vertical (e.g., Figure 2b) and the evaporation also has a certain direction which means the thinner the AAO template is, the more accurate the results will be (e.g., thick AAO will induce feature size loss during pattern replication in evaporation); (2) the surface of the AAO template is very rough while the silicon substrate is much more smooth which makes it easier for thinner AAO to touch the substrate fully [25]; (3) if the AAO film is too thin, it can easily be damaged during the operation. Due to these above reasons, a ~500 nm-thick through-hole AAO was used as the initial template in the experiment. After evaporation, the AAO template was torn off with tape, in Figure 2c, the white film in the lower right corner between the cracks is AAO thin film that waiting to be torn off and the black circle is Al. As shown in Figure 2c, the period of Al is around 467 nm and the aperture is around 357 nm; the dimensions of Al circles match well with those of the AAO nanoholes, which demonstrates a success in using the AAO thin film replication process. Figure 2d shows the SEM top-view of the large area uniform Al cylinder arrays in low magnification; the measurement area is around 30 μm × 30 μm. Figure 2e is the cross-sectional image of the Al arrays, the measured height of Al is around 99 nm. Figure 2f–i are the SEM images of AAO nanostructured silicon, in which Figure 2f,h are the low magnification images, Figure 2g,i are the high magnification images. As shown, the final particular structure is like putting countless cups together, so we label it CSNP. One can notice that the CSNP Si arrays are formed on the whole surface without obvious drawbacks, which proves the validity of the proposed replication method.

Although the thin film AAO scheme can, in large part, retain the feature size (inherited from the AAO nano-hole) of the evaporated material, the diameter of the Al nano-pillar decreases as the evaporation continues, which finally leaves the circular truncated cone-shaped (rather than standard cylinder) Al nano-pillar on the surface of silicon as shown in Figure 2e. It turns out that the aperture size of the AAO nano-hole shrinks as the thickness of the deposited Al increases, which can be explained by the side-wall growth effect. During the evaporation, the side-wall surface of the AAO template will also be deposited, so the thicker the deposited Al is, the smaller the AAO nano-hole diameter will be, which consequently induces the circular truncated cone-shaped Al arrays.

**Figure 3:**
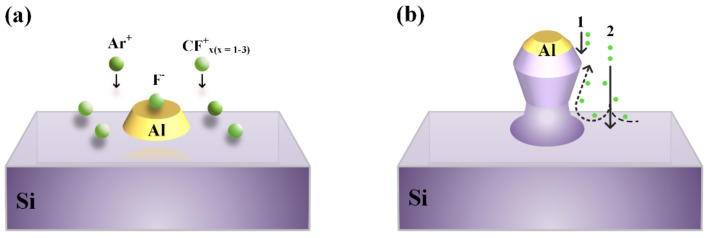
Schematic diagram for the formation of the cup-shaped Si nano-pillar arrays. (**a**) Al cylinder on the Si substrate at the beginning of RIE dry etching, (**b**) RIE dry etching mechanism for cup-shaped Si nano-pillar structures (1: the formation of the mouth of the cup, 2: the formation of the body of the cup).

Figure 3 shows the surface morphology before and after RIE dry etching. In Figure 3a, the reactive ion etching with Ar and CF_4_ gas plasma was used to bombard the surface of Al nanostructure on Si. In order to ionize a gas molecule or atom to form plasma, etching gas must be used to produce glow discharge under the function of high frequency electric field. In plasma, there are positive ions (Ion^+^), negative ions (Ion^−^), radicals, and free electrons, and the active radicals are responsible for making chemical reaction with etched material. In this experiment, the positive ions (Ar^+^, F^+^, CF^+^, CF_2_^+^, CF_3_^+^), the negative ions (F^−^, CF_3_^−^) and the radicals (F, F_2_, CF, CF_2_, CF_3_) are included [42,43,44]. The main reaction that takes place is as follows:

(1) $${\mathrm{CF}}_{4}+e^{-}\to {\mathrm{CF}}_{3}^{+}+F+{2e}^{-}$$

(2) $$4F+\mathrm{Si}\to {\mathrm{SiF}}_{4}$$

Surface morphology after RIE is shown in Figure 3b; the formation process of the cup-shaped Si nano-pillar can be explained by two different processes: the first one is the circular truncated cone-shaped Si formation illustrated by number 1 in Figure 2b. When the plasma hits the surface, both the Al and Si are etched and the size of the Al narrows down as the etching goes on. Therefore, the outer edge of upper silicon is etched more deeply than the inner edge, which leads to the bevel of the upper edge of the silicon. As a benefit from a high etching selectivity ratio of Al to Si (around 1:10), the formed Si nanostructure will have the similar morphology as the Al mask but stretched in the vertical direction. This process helps to form the mouth of the cups. At the same time, the second type of etching process takes place, as illustrated by number 2 in Figure 2b. As shown, the surface without the cover of the Al mask will be bombarded freely and the plasma flow will turn around when they reach the bottom, some of them will turn left while some turn right, which induces an obvious lateral etching effect. As the height increases, the speed and density of the plasma flow decreases, thus formed the body of the cups. These two processes combined to make the surface morphology of the CSNP.

### 3.2. Anti-Reflection Properties

**Figure 4:**
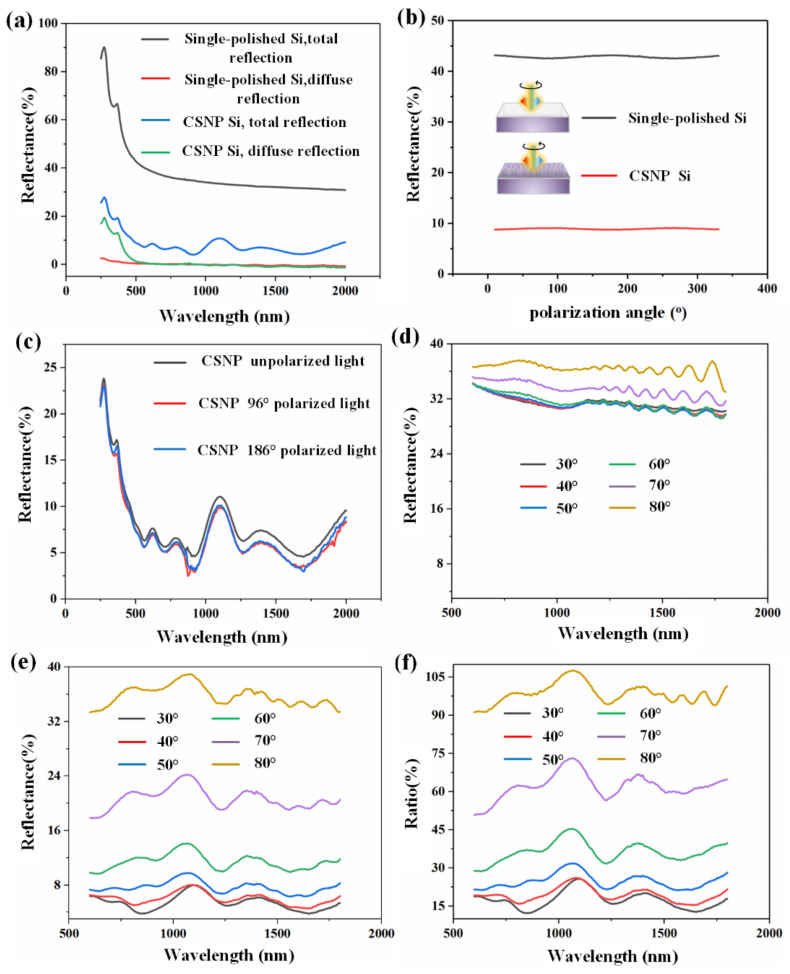
Reflectance measurement curves: (**a**) the curve of transmittance versus wavelength for the total reflection and diffuse reflection of single-polished Si and CSNP Si. (**b**) polarization-dependent measurements of the total reflection for single-polished Si and CSNP Si, with a fixed incident light wavelength of 500 nm, inserts illuminate the corresponding samples (**c**) wavelength-dependent total reflection measurements of the CSNP Si. (**d**) wavelength dependent total reflection measurements of the single-polished Si, with incident angles between 30°–80°. (**e**) wavelength dependent total reflection measurements of the CSNP Si, with incident angles between 30°–80°. (**f**) reflectance ratio calculated by using data from (**e**) divided by that of (**d**). (**a**–**c**) are the normal incidence measurements with a standard 8° incident angle.

So far, the application potential of AAO nanostructures has been proven in various high-performance components and devices [45,46,47]. Here, the AR properties of the fabricated CSNP Si have been studied through numerous parameters, namely wavelength, polarization incident angle, etc., in order to reveal their basic working mechanism, as shown in Figure 4. From Figure 4a, one can easily observe a sharp decline when comparing the total reflection of CSNP Si to the single-polished Si in a measurement wavelength range of 250 nm to 2000 nm. Specifically, the decrement increases for a shorter wavelength and reaches around 60% at 250 nm, and even for the long wavelength range, the reflectance drops from above 30% to below 10%, which proves the excellent AR performance of the CSNP structures. Another interesting phenomenon is that when wavelength exceeds 550 nm, the reflectances of diffuse reflection for single-polished Si and CSNP Si overlap each other, which means that there are only mirror reflections for both samples in longer wavelength. Actually, this phenomenon can be explained by equivalent medium theory (or sub-wavelength structure effect). In short, when *D* < *λ*/*n_s_*, where *D* is the dimension of a structure, *λ* is the light wavelength and *n_s_* is the refractive index of the substrate, the light cannot distinguish the structure and, instead, will consider it as a uniform material with equivalent refractive index *n_eff_* defined by [17,24]:

(3) $$(1-f)\frac{n_{s}^{2}-n_{i}^{2}}{n_{s}^{2}-n_{eff}^{2}}=\frac{n_{i}}{n_{eff}}$$

where *n_i_* indicates the refractive index of the surrounding materials and *f* is the duty cycle of structures. Consequently, the CSNP will act like a uniform layer and weaken the diffuse reflection (induced from structure diffraction, scattering, etc.) in the long wavelength range as shown in Figure 4a. Moreover, for a surface thin film, the *λ*/4 thin film theory is commonly used for AR performance optimization [17,24]:

(4) $$n_{eff}h=\frac{(2m+1)\lambda }{4}(m=0,1,2,3\cdots )$$

where *h* defines the thickness of the thin film. Thus, the reflectance curve of the total reflection for CSNP Si oscillates with the wavelength in the long wavelength (as shown in Figure 4d) and reaches minimum when a specific m is satisfied in Equation (4). There are two commonly used methods for AR optimization based on equivalent refractive index defined by Equation (3). The first one is to acquire a desired *f* and let the *n_eff_* satisfy *n_eff_* = √*n_i_n_s_*, which is applicable for the vertical side-wall profile situation [17,24]. The other one is to tailor the side-wall profile of the nano-pillar to make a gradually changed equivalent refractive index along the pillar direction [27], like the CSNP structure proposed in this work. Figure 4b shows the polarization-dependent angle reflectance curves; no obvious polarization dependent properties can be seen for both single-polished Si and CSNP Si. The 96° and 186° polarized lights are used for the study of cross polarization performance in a wide band as shown in Figure 4c, again, no obvious polarization-dependent properties can be detected in the whole measured wavelength band ranges from 250 nm to 2000 nm. The above results indicate that the as-fabricated CSNP nanostructure is insensitive to the polarization angle, which is of great importance to the application area with polarization-independent requirements [48,49,50,51]. Figure 4e gives the incidence angle-dependent performance of the CSNP Si; as shown, the reflectance increases significantly as the incidence angle increases. In addition, similar oscillation can be observed in Figure 4d because of the reflection from the back side of the Si. The oscillation of the reflectance intensifies as the wavelength increases; this is because the absorption (imaginary part of the refractive index) of Si becomes smaller for a longer wavelength, which means that more light will return back from the back side of Si and participants into the interference at the front surface. Actually, similar oscillation exists in all curves of Figure 4 if one zooms into these figures to some extent. Figure 4e shows the same incidence angle-dependent tendency as that of Figure 4d, but with a relatively low reflectance, thanks to the excellent AR performance of the CSNP nanostructures. The result using data from (e) divided by that of (d) is shown in the Figure 4f, which represents the reflectance ratio. As shown, the CSNP nanostructure shows a very good AR advantage in a small incidence angle, and the reflectance ratio reaches around 15% under 30° irradiation, which is of great use for high performance optoelectronic devices and encourages us to further study the light-trapping property of the CSNP structured solar cells.

### 3.3. Analysis and Simulations

**Figure 5:**
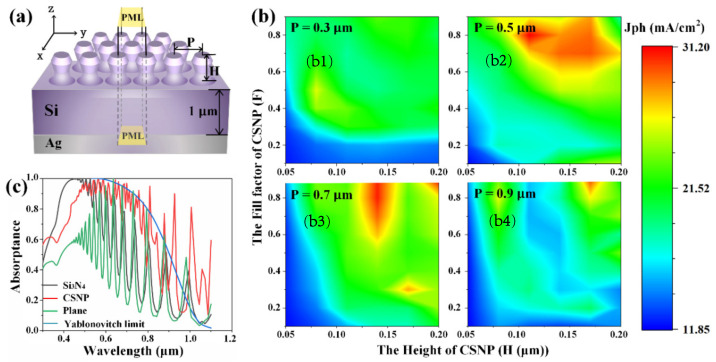
The simulation of a solar cell: (**a**) Simulation model of the CSNP thin film Si solar cell. (**b**) Relationships between the parameters of CSNP and J_ph_ of solar cell at different periods for (**b1**) 0.3 μm, (**b2**) 0.5 μm, (**b3**) 0.7 μm, and (**b4**) 0.9 μm. Height ranges from 0.05 μm to 0.2 μm with a step size of 0.03 μm. Fill factor ranges from 0.1 to 0.9 with a step size of 0.1. (**c**) Absorptance calculation results for 1 μm thick solar cells under different architectures. CSNP with P = 0.5 μm, F = 0.8 and H = 0.11 μm was used and the thickness of Si_3_N_4_ has been optimized. The absorptance of Yablonovitch light capture limit of 1 μm thick c-Si was used for reference.

There are two important aspects regarding the optical performance of a high-quality quantum efficiency optoelectronic device; one is the AR property from a surface, and the other one is the capture ability for the entered light. Both of these two aspects have been extensively studied in the field of nanostructured solar cells, especially for thin film solar cells [13,52,53,54], and have usually been called the light-trapping properties. In order to study the light trapping performance of the CSNP, a silicon-based thin film solar cell model has been built as shown in Figure 5a, in which the symmetrical two adjacent triangular lattices serve as the smallest simulation area [23]. This simulation model can be divided into three layers: the bottom layer is Ag, which is a metal back electrode, the middle layer is Si which is the main body of the solar cell, and the top layer is the CSNP which helps to trap light. In the simulation, under 0-degree and 90-degree polarizations, photocurrent density (J_ph_) was calculated, and the plane wave source ranging from 0.3 μm to 1.1 μm was used. The results of the unpolarized sunlight are defined by the average of these two polarizations. In brief, when the light source is 0-polarization (x polarization direction), it has asymmetric boundary conditions in the x direction and symmetric in the y direction. On the contrary, when the light source is 90-polarization (y polarization direction), it has symmetric boundary conditions in the x direction and asymmetric boundaries in the y direction, which can significantly reduce the simulation memory and time. In the z direction, perfectly matched layer (PML) boundary conditions were applied. The period and height of the CSNP have been marked in the picture with a 1 μm-thick Si underneath. The calculation of J_ph_ represents the quantitative measurement of the nanostructured solar cell light-trapping ability [55]. Under the condition of solar radiation AM1.5, the J_ph_ can be obtained by the following formula [27,28]:

(5) $$J_{\mathrm{ph}}=e\int_{300nm}^{1100nm}\frac{\lambda }{hc}A(\lambda )I_{AM1.5}(\lambda )d\lambda$$

(6) A(λ)=1−R(λ)−T(λ)

where *e* is the electron charge, *h* is the Planck constant, *c* is the speed of light in vacuum, I_AM1.5_(*λ*) is the incident light spectrum AM1.5, A(*λ*) represents the absorption of solar energy by Si, R(*λ*) represents the reflection, and T(*λ*) represents the transmission.

In the simulation, the rule between structural parameters and J_ph_ emerges by varying P (Period), H (Height), and F (Fill factor), which helps to study the light-trapping performance of CSNP. The results are shown in Figure 5b. As shown, the parameters of the CSNP applied can have a great impact on the results of J_ph_. When the period changes from 0.3 μm to 0.9 μm, the change of J_ph_ (as the increase of period) displays the following tendencies: (1) the structural parameters for optimal value of the J_ph_ shows a positive dependency as H and F increases, respectively; (2) the optimized J_ph_ increases first and then drops with a maximum value 31.2 mA/cm^2^ obtained at P = 0.5 μm. 

In order to establish the light trapping performance of CSNP, Table 1 lists the optimized J_ph_ with different structural parameters of the solar cells in this study and those of recent literature as a reference [27]. The same parameters, such as device model, structure height, period, and fill factor, have been studied in the reference work so the comparison can be applicable. As shown in Table 1, the maximum value of J_ph_ (31.2 mA/cm^2^) for CSNP solar cell is obtained at P = 0.5 μm, H = 0.11 μm and F = 0.8, which shows obvious improvement comparing with that of the cylinder (27.95 mA/cm^2^), cone (25.75 mA/cm^2^) and parabola (28.45 mA/cm^2^) structured ones. Specifically, comparing with the optimized Si_3_N_4_ thin film solar cell, an increment of 54.64% has been achieved for the CSNP thin film solar cell, which shows a great potential for the application of CSNP and its corresponding components and devices.

According to Equation (5), the increase of J_ph_ represents a strong absorption. Figure 5c displays the absorptance of the solar cell with CSNP, planar c-Si and Si_3_N_4_ surface coating. The Yablonovitch limit is expressed as [56,57]:

(7) $$A_{b}=1-\frac{1}{1+4n^{2}\alpha d}$$

where *n* is the real part of the refractive index of the material, *α* is the absorption coefficient, and d is the thickness of the absorption layer. The absorptance of solar cells can be significantly enhanced as shown in Figure 5c due to the multiple effects including destructive wave interference, total internal reflection, and scattering introduced by surface nanostructure. The CSNP solar cell shows a much higher absorptance over the whole wavelength ranges from 0.3–1.1 μm when comparing with the plane solar cell, but a slightly lower value than that of the Si_3_N_4_ solar cell at wavelengths around 0.35–0.5 μm. In fact, our previous study shows that one can improve the short wavelength behavior of a thin film solar cell by reducing the period of surface nanostructure and combining it with a bottom metal grating to optimize the whole wavelength band performance [27,28]. In addition, one may notice that the curve of the CSNP solar cells shows an obvious raising step at around 0.43 μm in Figure 5c. This sharp step is only related to the period and is induced from the first-order diffraction [27]. Moreover, when light wavelength is greater than around 0.45 μm, a resonance of the spectrum can be easily observed at every curve, in Figure 5c. The reason behind this is the fact that a strong absorption coefficient belongs to the silicon only when the wavelength is relatively shorter, which ensures that the light that enters is totally absorbed with no reflectance. However, when it comes to a longer wavelength, the light that enters has the chance to be reflected back and interferes at the front surface. Consequently, due to the excellent light trapping property of the CSNP, the resonance peaks induced breaks the Yablonovitch limit at the wavelength range of 0.64–1.1 μm.

**Figure 6:**
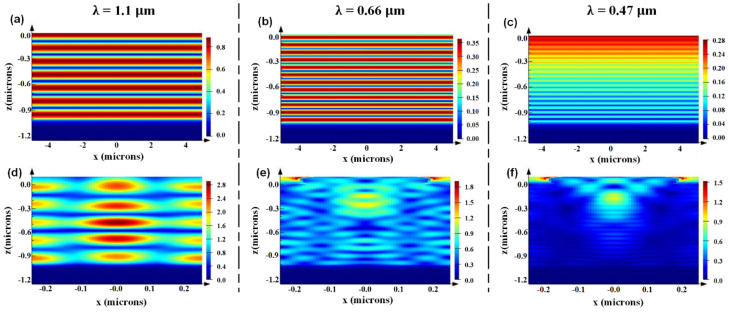
At normal incidence, the electric field distribution |E| at the X-Z cut of the plane solar cell for (**a**–**c**), CSNP solar cell for (**d**–**f**). The wavelengths are fixed at 1.1 μm, 0.66 μm and 0.47 μm (from left to right).

The distribution of electric field strength is shown in Figure 6. The mechanism of light capture ability of plane and CSNP solar cells were studied by their electric field at 1.1 μm, 0.66 μm and 0.47 μm. Regarding Si-based solar cells, there is a negative correlation between absorption coefficient and wavelength, as outlined above, so the absorption is relatively stronger at shorter wavelengths, which can be easily observed by comparing Figure 6a–c (or Figure 6d–f). According to the equivalent medium theory, for a long light wavelength like 1.1 μm, the light cannot distinguish the structure and will consider it as a uniform material with equivalent refractive index *n_eff_* defined by Equation (3). Thus, the propagation direction of the light will not be changed, as proven by Figure 6a,d with similar electric filed distributions, which corresponds to the leaky mode and shows little help for the light-trapping enhancement. With respect to the shorter wavelengths, like 0.66 μm and 0.47 μm, the diffraction effect dominants and the diffraction angle for a normal incidence can be defined by [28]:

(8) Psinθ=mλ(m=±1,±2,±3…)

where *P* represents the period of the nanostructure, *θ* defines the diffraction angle, *m* is the diffraction order, and *λ* is the light wavelength in materials. Due to the diffraction effect of CSNP, the incidence light changes its propagation direction to form a longer light path or even a guided mode, which greatly enhances the light absorption as shown by Figure 6e,f. It should be noticed that, although the guided mode disappears under a long wavelength, the enhanced Mie scattering resonance and the F-P resonance still exist [27], thus ensuring an enhanced absorption as shown Figure 5c and Figure 6.

## 4. Conclusions

In summary, we used the depositing method to successfully realize the uniform pattern transfer via a 500 nm-thick AAO membrane, followed by RIE dry etching, and free standing CSNP Si arrays were obtained at large scale. The CSNP-nanostructured Si showed excellent AR performance when compared with that of the single-polished Si over the whole spectrum ranges from 250 nm to 2000 nm. The CSNP Si arrays were applied to a thin film solar cell simulation model based on FDTD method for further study of the light trapping effect of the CSNP nanostructures. The J_ph_ of the CSNP thin film solar cell (obtained from simulations) showed an even higher value (31.19 mA/cm^2^) than that of the cylinder (27.95 mA/cm^2^), cone (25.75 mA/cm^2^), and parabola (28.45 mA/cm^2^) structured ones referenced from previous literature. The electric field distribution and the absorption curves containing Si_3_N_4_ coating, the CSNP, plane Si, and the Yablonovitch limit further established the high-performance mechanism of the CSNP. These results suggest that the CSNP nanostructure could be a good alternative for high-performance light-trapping applications.

## Figures and Tables

**Table 1:** Maximum J_ph_ of Nanostructured Solar Cells.

Nanostructure-Type	Fill Factor	Height (μm)	Period (μm)	J_ph_ of Maximum (mA/cm^2^)
Si_3_N_4_	-	0.06	-	20.17
CSNP	0.8	0.11	0.5	31.19
Parabola [27] Cone [27] Cylinder [27]	0.74 0.9 0.38	0.2 0.2 0.11	0.5 0.5 0.5	28.45 25.75 27.95

## Data Availability

The data presented in this study are available on request from the corresponding author.
